# Growth Hormone Treatment Promotes Remote Hippocampal Plasticity after Experimental Cortical Stroke

**DOI:** 10.3390/ijms21124563

**Published:** 2020-06-26

**Authors:** Sonia Sanchez-Bezanilla, N. David Åberg, Patricia Crock, Frederick R. Walker, Michael Nilsson, Jörgen Isgaard, Lin Kooi Ong

**Affiliations:** 1 School of Biomedical Sciences and Pharmacy and the Priority Research Centre for Stroke and Brain Injury, The University of Newcastle, University Dr, Callaghan, NSW 2308, Australia; sonia.sanchezbezanilla@uon.edu.au (S.S.-B.); rohan.walker@newcastle.edu.au (F.R.W.); michael.nilsson@newcastle.edu.au (M.N.); 2 Hunter Medical Research Institute, Lot 1, Kookaburra Cct, New Lambton Heights, NSW 2305, Australia; patricia.crock@newcastle.edu.au; 3 Institute of Medicine, The Sahlgrenska Academy, University of Gothenburg, 40530 Gothenburg, Sweden; david.aberg@medic.gu.se; 4 Department of Internal Medicine, Region Västra Götaland, Sahlgrenska University Hospital, Blå stråket 5, 41345 Gothenburg, Sweden; 5 Department of Paediatric Endocrinology and Diabetes, John Hunter Children’s Hospital, Kookaburra Cct, New Lambton Heights, NSW 2305, Australia; 6 Priority Research Centre GrowUpWell, The University of Newcastle, University Dr, Callaghan, NSW 2308, Australia; 7 NHMRC Centre of Research Excellence Stroke Rehabilitation and Brain Recovery, Heidelberg, VIC 3084, Australia; 8 Centre for Rehab Innovations, The University of Newcastle, NSW 2305, Australia; 9 LKC School of Medicine, Nanyang Technological University, 50 Nanyang Ave, Singapore 639798, Singapore; 10 School of Pharmacy, Monash University Malaysia, Bandar Sunway, Subang Jaya 47500, Selangor, Malaysia

**Keywords:** cognition, growth hormone, hippocampus, neurogenesis, synaptic plasticity, visual discrimination

## Abstract

Cognitive impairment is common after stroke, and disturbances in hippocampal function are often involved, even in remote non-hippocampal injuries. In terms of hippocampal function, growth hormone (GH) is known to affects plasticity and cognition. We aimed to investigate whether GH treatment after an experimental cortical stroke could enhance remote hippocampal plasticity and the hippocampal-dependent visual discrimination task. C57BL6 male mice were subjected to cortical photothrombotic stroke. Stroke mice were then treated with either saline or GH at 48 h after occlusion for 28 days. We assessed learning and memory using mouse touchscreen platform for the visual discrimination task. We also evaluated markers of neural progenitor cells, synaptic plasticity and cerebrovascular remodelling in the hippocampal formation. GH treatment significantly improved the performance on visual discrimination task after stroke. We observed a concomitant increased number of bromodeoxyuridine-positive cells in the dentate gyrus of the hippocampus. We also detected increased protein levels and density of doublecortin, a neuronal precursor cells marker, as well as glutamate receptor 1 (GLuR1), a synaptic marker. These findings provide further neurobiological evidence for how GH treatment could be used to promote hippocampal plasticity in a remote region from the initial cortical injury, and thus enhance cognitive recovery after stroke.

## 1. Introduction

Stroke leads to cognitive impairment partly due to the disruption of hippocampal function. Recent studies provided evidence that stroke causes hippocampal structural changes and dysregulation of hippocampal networks, leading to memory decline in humans [1,2,3,4,5]. Interestingly, cerebral ischemia has been shown to stimulate the proliferation of endogenous neural progenitor cells in animal models [6] and stroke patients [7]. Previous studies have suggested that ischemic stroke triggers increased proliferation of neural progenitor cells in the hippocampal subgranular zone [8,9,10,11,12,13,14], and the migration of these cells to the damaged areas [15,16,17,18]. However, these potential self-repair mechanisms are thought to operate only for a restricted time period after stroke, with the number of newborn neurons being insufficient for full tissue repair and their existence being transitory [19]. Recent findings suggest that a significant portion of newborn neurons generated after stroke reveal aberrant morphology and fail to correctly integrate into pre-existing networks [20,21]. Given that the capacity of this regenerative mechanism is limited, a therapeutic intervention that can stimulate neurogenesis and promote the integration of newborn neurons would be highly desirable to promote post-stroke recovery of cognition.

Interestingly, the pro-cognitive effects of growth hormone (GH) have been demonstrated in multiple neurological conditions, including traumatic brain injury [22]. GH has been shown to stimulate the genesis of neurons and endothelial cells, as well as to promote myelination and synaptogenesis [23]. Importantly, we have recently shown that GH treatment after photothrombotic vascular occlusion of the somatosensory and motor cortex promoted a marked improvement in the performance of associative memory cognitive domain as measured by the paired-associate learning task [24]. We additionally found an enhancement of neurorestorative processes, such as increased cell proliferation, neurogenesis, increased synaptic plasticity, myelination and angiogenesis within the peri-infarct region, most likely contributing to the improvement in motor outcomes [24,25,26,27,28]. However, we did not investigate the pro-cognitive effects of GH treatment on other cognitive domains and possible neurorestorative changes in the hippocampus. Early studies by Pathipati at al. demonstrated that intracerebroventricular delivery of GH after experimental stroke improved spatial memory using the Morris water maze test; however, they did not investigate changes in the hippocampus [29]. Thus, there are limited studies documenting the role of GH on hippocampal plasticity in the context of stroke recovery.

Therefore, an important question that we wanted to address in the current study was how GH treatment could enhance neurorestorative processes within the hippocampus remote to a cortical stroke, thus leading to an improvement in overall post-stroke cognition. Specifically, we examined changes in the density, distribution and protein expression of markers of neural progenitor cells in the hippocampal formation, using bromodeoxyuridine (BrdU) tagging and doublecortin (DCX). We also examined changes in the AMPA receptor subunit glutamate receptor 1 (GluR1), which plays an important role in synaptic plasticity. In addition, we examined changes in cerebrovascular density, using tomato lectin, a blood vessel stain. We assessed the effect of GH treatment on learning and memory using a mouse touchscreen platform for the hippocampal-dependent visual discrimination (VD) task [30,31].

## 2. Results

### 2.1. GH Treatment Improves Cognitive Function

**Figure 1:**
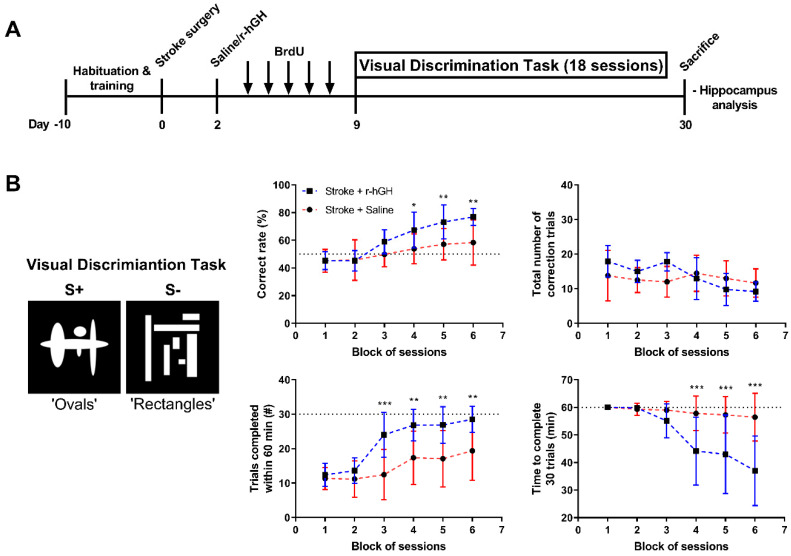
(**A**) Experimental design. (**B**) Illustration of the visual discrimination (VD) task. Images represent the pair of stimuli used for the task (‘ovals-rectangles’ pair) (S+ correct and S− incorrect). A variety of metrics were measured to assess cognitive performance in mice treated with recombinant human growth factor (r-hGH) compared to saline. Mean ± SD (two-way ANOVA and Sidak’s multiple comparisons). * *p* < 0.05; ** *p* < 0.01 and *** *p* < 0.001.

Cognitive function was assessed by hippocampal-dependent VD task for 18 consecutive days (Figure 1). We found a significant increase in % correct rate in r-hGH–treated stroke mice compared with saline (F_(1,18)_ = 7.402, *p* = 0.014), and a significant time effect (F_(5,90)_ = 27.80, *p* < 0.0001). Post hoc analysis indicated an increase in the % of correct rate in the fourth, fifth and sixth blocks of sessions in r-hGH–treated stroke mice (13.54%, *p* = 0.036; 16.12%, *p* = 0.007 and 18.52%, *p* = 0.001, respectively). We also observed a significant increase in the number of trials completed within 60 min (F_(1,18)_ = 13.00, *p* = 0.002), and decrease in the time required to complete 30 trials (F_(1,18)_ = 10.35, *p* = 0.005) in r-hGH–treated stroke mice (Figure 1B). Post hoc analysis indicated an increase in the number of trials completed within 60 min from the third to sixth block of sessions (11.60%, *p* = 0.0002; 9.47%, *p* = 0.0033; 9.80%, *p* = 0.0022; 9.10%, *p* = 0.0053), and a decrease in the time required to complete 30 trials from the fourth to sixth blocks of sessions (13.67%, *p* = 0.0009; 14.28%, *p* = 0.0005; 19.39%, *p* < 0.0001) in r-hGH–treated stroke mice.

Pearson correlation analysis shows a significant correlation between plasma insulin-like growth factor 1 (IGF-1) levels and the performance of hippocampal-dependent VD task at the final session; percentage correct rate (*r* = 0.4492; P _(Y = 0.1102X + 30.84)_ = 0.0469), trials completed within 60 min (*r* = 0.4536; P _(Y = 0.04704X + 7.385)_ = 0.0446), and time to complete 30 trials (*r* = −0.6186; P _(Y = −0.1180X + 87.70)_ = 0.0036).

### 2.2. GH Treatment Promotes Cell Proliferation and Neurogenesis in the Dentate Gyrus (DG)

**Figure 2:**
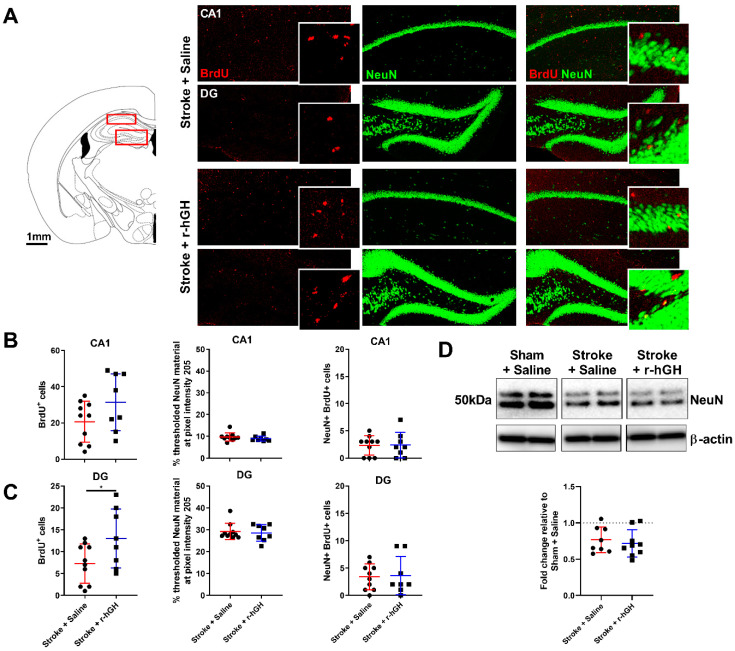
(**A**) Left panel: Schematic picture illustrating the location of the hippocampus region examined (CA1 and dentate gyrus (DG)). Right panel: Representative immunofluorescence images of bromodeoxyuridine (BrdU; red) and neuronal nuclei (NeuN; green) (scale bar = 100 μm). (**B** and **C**) Quantification of BrdU-positive cells, thresholded NeuN material and BrdU-NeuN-positive cells in the CA1 and DG. (**D**) Representative Western blot and quantification of NeuN protein levels in the hippocampus. For full immunoblots, see Figure S1. Mean ± SD (2-tailed *t*-test). * *p* < 0.05.

**Figure 3:**
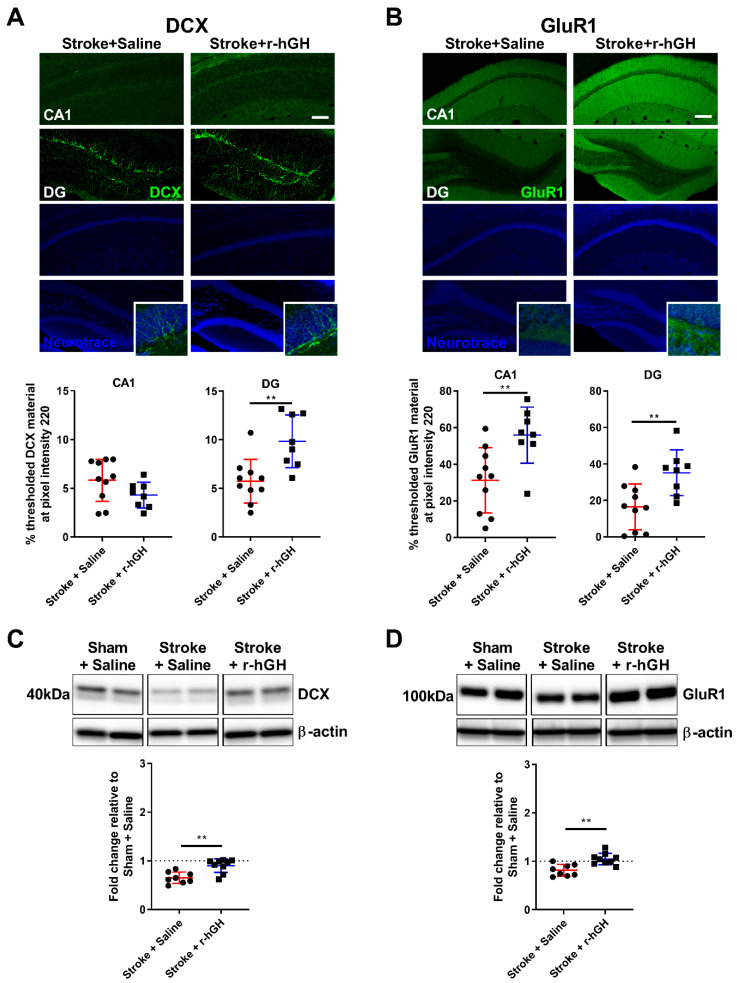
(**A**) Representative immunofluorescence images and quantification of doublecortin (DCX; green) in the CA1 and DG (scale bar = 100 μm). (**B**) Representative immunofluorescence images and quantification of glutamate receptor 1 (GluR1; green) in the CA1 and DG (scale bar = 100 μm). (**C**,**D**) Representative western blot and quantification of DCX and GluR1 protein levels in the hippocampus. For full immunoblots, see Figure S1. Mean ±S D (2-tailed *t*-test). ** *p* < 0.01.

We assessed BrdU/NeuN co-labelling and doublecortin (DCX) levels in two different sub-regions of the hippocampal formation: CA1 and DG (Figure 2A). r-hGH treatment significantly increased the number of BrdU-positive cells in the DG (78.1%, *p* = 0.004, Figure 2B), but not in the CA1 (Figure 2C). There were no differences in area of thresholded material for NeuN, NeuN protein levels and the number of BrdU-NeuN-positive cells (Figure 2). r-hGH treatment significantly increased the material thresholded for DCX in the DG (82.0%, *p* = 0.002, Figure 3A). We also found a significant increase in DCX protein levels (0.25-fold, *p* = 0.001; Figure 3C) in r-hGH–treated stroke mice.

### 2.3. GH Treatment Promotes Expression of GluR1 within the Hippocampal Formation

We performed immunofluorescence analysis for a synaptic marker (GluR1) in the hippocampal formation. The immunofluorescence data revealed a significant increase in material thresholded for GluR1 in the CA1 (79.0%, *p* = 0.007, at pixel intensity 220) and DG (113.3%, *p* = 0.006, at pixel intensity 220) in r-hGH–treated stroke mice (Figure 3B). Further, an increase in GluR1 protein levels (0.24-fold, *p* = 0.001; Figure 3D) was found in r-hGH–treated stroke mice.

### 2.4. GH Treatment Had No Effect on the Formation of Cerebral Vasculature within the Hippocampal Formation

**Figure 4:**
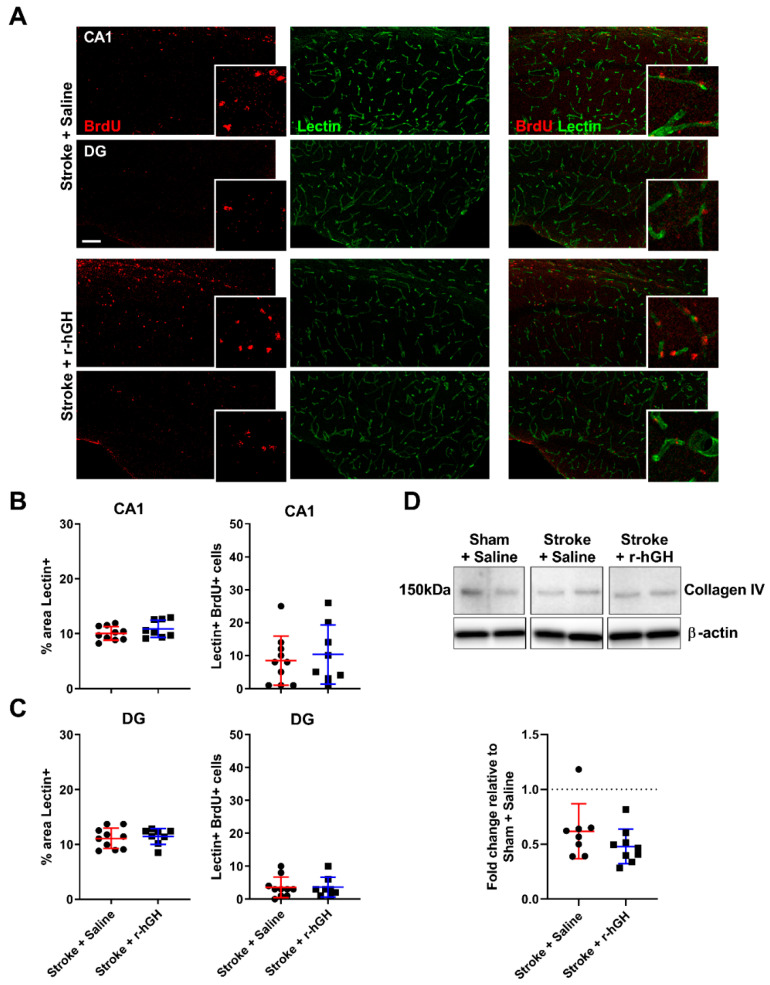
Analysis of cerebrovascular remodeling within the hippocampus region. (**A**) Representative immunofluorescence images of BrdU (red) and tomato lectin (green) and high magnification detail (scale bar = 100 μm) in the CA1 and DG subregion of the hippocampus. (**B**) Quantification of the number of BrdU-lectin-positive cells and % of area covered by tomato lectin in the CA1. (**C**) Quantification of the number of BrdU-lectin-positive cells and % of area covered by tomato lectin in the DG. There were no significant differences in any of the parameters between stroke mice treated with r-hGH and the saline group. (**D**) Representative western blot and Collagen IV protein levels in the hippocampus. For full immunoblots, see Figure S1. Mean ± SD.

We analyzed the density of the brain vasculature in the hippocampal formation using immunofluorescence labelled tomato lectin, which binds to glycoproteins located in the glycocalyx and in the basal membrane of endothelial cells. We observed no statistically significant differences in the area covered by tomato lectin and the number BrdU-lectin-positive cells in the hippocampal formation. Furthermore, no significant changes in Collagen IV protein levels in r-hGH–treated stroke mice (Figure 4).

### 2.5. GH Treatment Promotes Restoration of White Matter Disturbances

**Figure 5:**
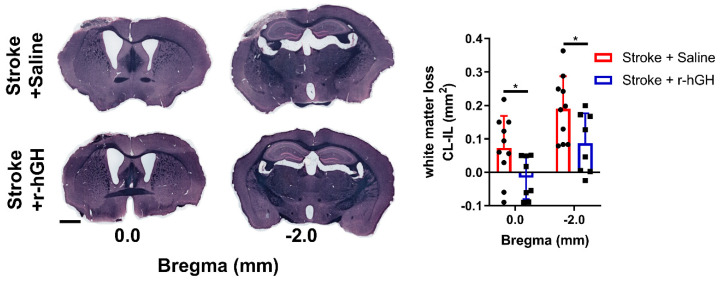
Sudan black staining of white matter tracts from the corpus callosum (Bregma 0.0 mm and −2.0 mm). White matter loss was calculated as contralateral hemisphere area—ipsilateral hemisphere area (mm^2^). Mean ± SD. * *p* < 0.05. Scale bar = 1 mm.

We evaluated white matter tract alterations in the corpus callosum using Sudan black staining of myelin at Bregma 0.0 mm and −2.0 mm. We found a significant decrease in white matter structural loss in corpus callosum at Bregma 0.0 mm (122.0%, *p* = 0.036) and Bregma −2.0 mm (54.4%, *p* = 0.035) at 30 days post-stroke in r-hGH–treated stroke mice (Figure 5).

## 3. Discussion

We demonstrated that GH treatment after experimental cortical stroke stimulates proliferation of neural progenitor cells and increases synaptic plasticity within the hippocampus, a remote region from the initial cortical injury, in parallel with improvements in hippocampal-dependent VD task. While long-term GH treatment after experimental stroke has been previously shown to improve cognitive function [24,29], this is the first study to our knowledge to comprehensively show both functional and hippocampal plasticity of GH treatment. Thus, the present study extends the understanding of how GH treatment acts on hippocampal plasticity to promote post-stroke cognitive recovery.

Our group recently established that cortical photothrombotic stroke impairs the ability of mice to discriminate between stimuli with a high degree of similarity [31]. Although our cortical stroke model does not cause a direct damage to the hippocampus, we observed an indirect significant decrease in the number of neurons within the hippocampus at 2 weeks post-stroke [31]. It should be noted that a cortical stroke could also induce a dysfunction in the hippocampal-thalamic network [32]. While cerebral ischemia causes persistent neuronal loss in remote regions that are functionally connected to the primary infarction site [33,34], interestingly, it also triggers a transient proliferation of endogenous neural progenitor cells in the DG of the hippocampus [6,35]. Importantly, we previously demonstrated that GH treatment after experimental stroke promotes the associative memory cognitive domain as measured by the paired-associate learning task [24]. However, we only investigated the neurorestorative effects of GH treatment within the peri-infarct regions [24]. The observed enhancement of recovery-promoting mechanisms within the peri-infarct area is most likely contributing to the improvement in motor outcomes [25], whereas the promotion of hippocampal neurogenesis and increased synaptic plasticity reported here are likely to be of importance for improved cognition.

The present study reports novel evidence that GH treatment promotes hippocampal neurogenesis, remotely from the cortical injury, and enhances the performance of hippocampal-dependent VD tasks. Our findings further suggest that higher levels of plasma IGF-1 are associated with better performance in hippocampal-dependent VD task, which is consistent with prior findings [24,36]. GH can exert its effects directly or indirectly via its mediator insulin-like growth factor 1 (IGF-1) on hippocampal plasticity [37]. Firstly, GH receptor expression is widespread in the brain and there are studies supporting a passage of GH over the blood–brain barrier; therefore, GH may have direct effects on the brain [38]. Secondly, it is possible for GH to increase production and secretion of local IGF-1 in the brain [39,40]. Thirdly, GH induces the secretion of IGF-1 in the circulatory system, and peripheral IGF-1 can penetrate the blood–brain barrier to exert effects on the brain [41,42]. However, it is not possible for our experimental design to specifically elucidate which mechanisms are involved. Nevertheless, we would suggest that it is most likely a combination of these pathways. We used BrdU tagging, to assess whether there is an increase in neural progenitor cells. We also evaluated the expression of DCX, a marker of immature neurons [43]. Our results show an increased number of BrdU-positive cells and expression of DCX in the DG subregion in GH-treated stroke mice. Together, these findings of increased neurogenesis are critical, as recovery of the cognitive function following stroke depends on cellular plasticity in the hippocampal formation [14,44].

We also investigated other key neurorestorative processes in the hippocampal formation or in its vicinity. Firstly, we assessed synaptic plasticity by measuring the AMPA receptor subunit GluR1, which plays an important role in synapse formation, stabilization, and plasticity [45,46,47]. We found that GluR1-positive structures and protein levels were increased in the hippocampus in GH-treated mice. While the results might be an indication of higher synapse numbers or higher synaptic activity, it should be noted that GluR1 may also be found extrasynaptically [48]. Future comprehensive investigation on synaptic changes using co-immunolabelling of synaptic structures and high-resolution microscopy is warranted. Secondly, given that stroke-induced remote secondary neurodegeneration is a disruption of connections between the cortex and hippocampus [31,32], we evaluated white matter tract disturbances. The results from Sudan black staining suggest that GH treatment provides restorative effects on white matter structural loss. Together, these results are particularly interesting when considered in conjunction with the cognitive function findings. Thirdly, we explored the effect of GH treatment on cerebrovascular remodeling. In our previous studies, we showed that GH has the ability to enhance angiogenesis, and increases the density and area coverage of both CD31 and Collagen IV–positive cells in the peri-infarct region [24,25]. In the current study, we analyzed vessel density in the hippocampus area using immunofluorescence labelled tomato lectin. However, GH treatment did not affect the % area covered by tomato lectin or the number of BrdU-lectin-positive cells in the hippocampal formation, as we have previously shown in the peri-infarct area. These results suggest that some effects of GH appear to be global in the brain, while others are more brain region-specific and cell type-specific.

There are two important points regarding the experimental design of the current study that require consideration. Firstly, we used r-hGH for practical purposes and from the translational point of view. r-hGH is much more accessible than mouse GH, and has been used in a wide setting of mouse experimental models with expected GH-related results. However, there some differences in the actions of r-hGH and mouse GH. Although r-hGH stimulates the GH receptor and an increase in serum IGF-I, there is also a potential minor stimulation of prolactin receptors [49,50]. Nonetheless, it appears that prolactin is not able to induce local IGF-I, at least not in the skeleton [51]. Therefore, this does not compromise the results, but gives an alternative mechanism. Secondly, we administered r-hGH subcutaneously via mini-osmotic pumps for 28 days after experimental stroke, as we accounted for the impact of stress due to repeated injections. However, continuous infusion of GH may disrupt the endogenous GH pulsatile secretion, and different modes of GH administration can have minor different effects on the brain [52,53]. This warrants further investigation using programmable pumps.

## 4. Conclusions

We have shown that GH treatment can enhance neurogenesis and synaptic plasticity in the hippocampus, a remote region from a cortical injury, thus leading to improvement in cognition after stroke. Promisingly, small clinical studies have identified that GH treatment ameliorates cognitive impairment in stroke patients [36,54]. Further, due to the high incidence of GH deficiency in stroke survivors [55,56], GH treatment may represent a useful therapeutic intervention for many patients during stroke recovery.

## 5. Materials and Methods

### 5.1. Animals

All animal experiments were approved by the University of Newcastle Animal Care and Ethics Committee (A-2014-432, 23 November 2015) and undertaken in accordance to the Animal Research: Reporting of In Vivo Experiments guidelines. C57BL/6 mice (male, 10 weeks old, *n* = 48) were obtained from the Animal Services Unit at the University of Newcastle. Mice were maintained in a 12:12h reverse light–dark cycle (lights on 19:00h). All procedures conducted in the dark phase.

### 5.2. Sample Size Calculation

Sample size was estimated using G*Power 3.1 software. Using previous and preliminary data [24,25], we obtained an effect size of *d* = 1.6. Allowing a type 1 error of 5%, *α* = 0.05, with the power of 80%, *β* = 0.2, we calculated a samples size of 8 animals per group.

### 5.3. Experimental Design

The experimental design of this study was as described in Figure 1A [24]. A total of 48 mice were used in this study. All the experimental groups are randomized, and all outcome analyses were performed in a blinded manner.

The first cohort of mice (*n* = 24) was used to assess cognitive function, and brains were collected for histological analysis. At day 0 (D0), all mice were subjected to photothrombotic occlusion. At D2, mice were randomly allocated to receive either r-hGH or saline at 1.4 mg/kg body weight per day subcutaneously via mini-osmotic pumps for 28 days (stroke + saline, *n* = 12, and stroke + r-hGH, *n* = 12). At D3, mice were injected with BrdU (50 mg/kg body weight) for 5 consecutive days. To evaluate cognitive function, mice were subjected to mouse touchscreen platform for visual discrimination (VD) task for 18 days (days 9–26 post-stroke). At D30, mice were euthanized and their brains were collected. Mice were excluded from the study if we histologically identified that the stroke had not occurred or malfunction of the mini-osmotic pumps. Within the saline group, one mouse had to be excluded due to post-surgery death and another one due to no stroke. Within the r-hGH group, we had to exclude one mouse due to pump malfunction and two mice due to a problem with the perfusion procedure.

The second cohort of mice (*n* = 24) was used for biochemical analysis. At D0, mice were subjected to photothrombotic occlusion or sham surgery (stroke, *n* = 18, and sham, *n* = 6). At D2, stroke mice received r-hGH or saline as described above (sham + saline, *n* = 6, stroke + saline, *n* = 8, and stroke + r-hGH, *n* = 10). At D30, mice were euthanized and their brains were collected for Western blotting. Within this second cohort, one mouse from the stroke + r-hGH group had to be excluded for not having a stroke.

### 5.4. Photothrombotic Occlusion

Photothrombotic occlusion was performed as described previously [31,57,58]. Briefly, mice were anesthetized by 2% isoflurane during surgical procedure on a temperature controlled (37 ± 1 °C) stereotaxic frame. Rose Bengal (200 μL, 10 mg/mL solution in sterile saline, Sigma-Aldrich, St. Louis, MO, USA) was injected intraperitoneally. At 8 min post-injection, the skull was exposed and illuminated for 15 min by a cold light source positioned at 2.2 mm left lateral of Bregma 0.0 mm. For the sham group, the Rose Bengal injection was substituted with 200 μL of sterile saline (0.9% NaCl, Pfizer, Sydney, NSW, Australia).

### 5.5. Mini-Osmotic Pump Placement

Mini-osmotic pump (Model 2004, Alzet, Cupertino, CA, USA) placement was performed as preciously described [24,25,59]. At D2, the mini-osmotic pumps were implanted. An incision was made in the skin between the scapulae where the mini-osmotic pump was inserted. The pumps were filled with 200 μL of either recombinant human growth hormone (r-hGH, Somatropin 10 mg/1.5 mL, SciTropin A, SciGen, Belrose, NSW, Australia) or sterile saline. The delivery rate of the pumps was 0.25 μL/h for 28 days (0.04 mg r-hGH per day).

### 5.6. Visual Discrimination (VD) Task

Mouse touchscreen operant chambers were used in the cognitive testing as described [30,31], and were conducted in a blinded and randomized manner. Briefly, mice were calorie restricted overnight before cognitive testing. Strawberry milkshake was used as a reward to motivate the performance of the mice. First, mice were trained to learn to associate a nose poke of the touchscreen and the delivery of the reward. Following training, mice underwent photothrombotic occlusion surgery. At D9, mice were subjected to the VD task. This task entailed simultaneous presentation of two stimuli: one correct (S+) and one incorrect (S−). The S+ stimulus appeared pseudo-randomly either in the right or left part of the touchscreen. When the mouse selected the correct choice, S+, a tone was triggered, the reward tray was illuminated, and strawberry milkshake was delivered to the tray. If the mouse selected the incorrect image, S−, there was no reward delivery, no tone, the house light was turned on for 5 s, and a correction trial was initiated. In each VD session, the testing ended once a mouse successfully completed 30 trials or reached a 60 min time limit, whichever occurred first. All mice were subjected to a total of 18 sessions.

### 5.7. Tissue Processing

For the first cohort, mice were anaesthetized with sodium pentobarbital. Mice were perfused via the ascending aorta with ice-cold 0.9% saline followed by ice-cold 4% paraformaldehyde (pH 7.4). Brains were dissected and post-fixed for 4 h in the same fixative then transferred to a 12.5% sucrose solution in 0.1M phosphate buffered saline (PBS) for storage and cryoprotection. Serial coronal sections were sliced on a freezing microtome (Leica, North Ryde, NSW, Australia) at a thickness of 30 μm.

For the second cohort, mice were anaesthetized with sodium pentobarbital, and transcardially perfused with ice cold 0.9% saline. Brains were dissected and rapidly frozen in −80 °C isopentane. Brain sections were sliced using a cryostat (−20 °C) at a thickness of 200 μm. The hippocampal formation (Bregma −1.2 to −2.5mm) samples were dissected and stored frozen in −80 °C until further analysis.

### 5.8. Immunofluorescence

Free-floating fixed sections were co-immunostained as previously described [60,61]. For BrdU staining, antigen retrieval was performed before the blocking step as follows: 10 min HCl (1M) incubation on ice, 10 min HCl (2M) incubation at room temperature, 20 min HCl (2M) incubation at 37 °C, 10 min borate buffer (0.1M) incubation at room temperature and three washes in PBS + 0.1% Triton X. Sections were blocked using 3% bovine serum albumin, then incubated with appropriate primary antibody (DCX, GluR1, BrdU, NeuN) overnight at 4 °C, and followed by 2 h incubation in corresponding secondary antibodies at 25 °C (see Table 1 for antibodies concentration). Tomato lectin staining was performed together with the secondary antibody incubation. NeuroTrace was used to counterstain sections after immunocytochemical detection of DCX or GluR1. Brain sections were washed with PBS in between each incubation step.

### 5.9. Sudan Black Staining

Sudan black staining was performed as previously described [62]. Briefly, sections were mounted and rinsed with 70% ethanol followed by 15 min incubation with Sudan Black B solution (Sigma-Aldrich, USA). After staining sections were rinsed with 70% ethanol and water and 5 min counterstained with nuclear fast red solution (Sigma-Aldrich, USA).

### 5.10. Image Acquisition and Analysis

Immunofluorescence high-resolution confocal images were taken on a Leica TCS SP8 confocal microscope with a Leica HC PLC APO 10 × 0.40 objective. For each region of interest, 30 μm z-stacks with a step size of 1 μm were taken. Imaging parameters (laser power, resolution and gain) were held constant throughout all imaging sessions. Exhaustive automated BrdU cell counts were performed using ImageJ software. For the analysis of NeuN, DCX and GluR1 labelling, we performed thresholding analyses and chose the optimal pixel intensity that clearly reflected the immunofluorescence signal. To measure vessel coverage (% lectin^+^ area), the emission channels were split and the lectin emission image was uniformly thresholded at a high stringency. The area of vessel coverage was expressed as a percentage of the overall field of view (ImageJ Software, National Institutes of Health, Bethesda, MD, USA). For BrdU/NeuN and BrdU/lectin co-labelling, we used the plugin ‘colocalization’ for ImageJ. This plugin highlights the colocalizated points of two 8-bits images. The colocalizated points appear black by default.

Sudan black images were acquired at 20× using Aperio AT2 (Leica, Wetzlar, Germany). Specifically, we focus on the corpus callosum and we assessed white matter loss as a difference between the contralateral and ipsilateral hemispheric area (mm^2^). The quantitative analysis was undertaken specifically in two sections (Bregma 0.0 and −2.0 mm).

### 5.11. Protein Extraction and Western Blotting

Protein extraction and Western blotting were performed as previously described [63,64]. Hippocampus samples were sonicated in 300 μL lysis buffer (50 mM tris(hydroxymethyl)aminomethane buffer pH 7.4 containing 80 μM ammoniummolybdate, 5 mM *β*-glycerolphosphate, 1 mM dithiothreitol, 1 mM ethylenediaminetetraacetic acid, 1% sodium dodecyl sulfate, 1 mM sodium pyrophosphate, 1 mM sodium vanadate, 1 cOmplete™ protease inhibitor cocktail tablet, and 1 PhosSTOP™ phosphatase inhibitor cocktail tablet) and centrifuged at 14000 *g* for 20 min at 4 °C. Supernatants were collected and protein concentrations were estimated by Pierce BCA protein assay kit (Thermo Fisher Scientific, Waltham, MA, USA). Sample buffer (2% sodium dodecyl sulfate, 50 mM tris(hydroxymethyl)aminomethane, 10% glycerol, 1% dithiothreitol, 0.1% bromophenol blue, pH 6.8) was added into the supernatants. To determine the protein levels of NeuN, DCX, GluR1 and Collagen IV, the protein homogenates from sham + saline (*n* = 6), stroke + saline (*n* = 8) and stroke + r-hGH (*n* = 9) were analyzed together on 26-well gels. Then, 15 μg of protein lysate was electrophoresed into Biorad Criterion TGX Stain-Free 4–20% gels and transferred to polyvinylidene fluoride membranes. The membranes were blocked with 5% skim milk in TBST for 1 h at room temperature and incubated overnight at 4 °C with the appropriate primary antibody: NeuN, DCX, GluR1 and Collagen IV (see Table 1 for antibodies concentration). The next day, membranes were incubated with secondary antibody for 1 h at 25 °C. In between each incubation step, membranes were washed in TBST. Chemiluminescence signals were detected on an Amersham Imager 600 using Luminata Classico western blotting detection reagent (Merck Millipore, Burlington, MA, USA). The density of the bands was measured using Amersham Imager 600 analysis software (GE Healthcare Life Sciences, Pittsburgh, PA, USA). The protein markers were normalized to *β*-actin (as loading control), and were expressed as a fold change of mean ± SD for each group relative to the mean of the sham + saline group.

### 5.12. Statistical Analyses

All data were expressed as mean ± SD and were analyzed using GraphPad Prism v7.02 (GraphPad Software, San Diego, CA, USA). The primary outcome measurement was differences between stroke + saline and stroke + r-hGH. Data from Western blotting, immunofluorescence labelling and white matter loss were analyzed using the 2-tailed *t*-test. VD task (18 sessions temporal analysis) was analyzed using 2-way ANOVA, followed by Sidak multiple comparisons. A *p* value < 0.05 was considered statistically significant.

## Figures and Tables

**Table 1:** List of antibodies used for western blot and immunofluorescence analyses.

Targets	Sources of Antibodies	Application	Dilution
BrdU	Sigma-Aldrich, mouse anti-BrdU, #B8434	IF	1:1000
NeuN	Cell Signaling, rabbit anti-NeuN (D3S31), #12943	WB	1:2000
		IF	1:1000
DCX	abcam, rabbit anti-doublecortin, #ab18723	WB	1:1000
		IF	1:1000
GluR1	Cell Signaling, rabbit anti-AMPA Receptor 1 (GluA1), #13185	WB	1:2000
		IF	1:1000
Collagen IV	Abcam, rabbit anti-collagen IV, #ab6586	WB	1:1000
β-actin	Sigma-Aldrich, Monoclonal Anti-β-actin-HRP antibody, A3854	WB	1:50,000
NeuroTrace	ThermoFisher Scientific, NeuroTrace™ 640/660 Deep-Red Fluorescent Nissl Stain, #N21483	IF	1:1000
Tomato Lectin	Vector Laboratories, DyLight 649 Lycopersicon esculentum (Tomato) Lectin #DL-1178	IF	1:1000
Rabbit IgG	Biorad, Anti-Rabbit-HRP antibody, #170-6515	WB	1:7500
	ThermoFisher Scientific, anti-Rabbit IgG (H+L) Highly Cross-Adsorbed Secondary Antibody, Alexa Fluor 488, #A21206	IF	1:400
Mouse IgG	Biorad, Anti-Mouse-HRP antibody, #170-6516	WB	1:10,000
	ThermoFisher Scientific, anti-Mouse IgG (H+L) Highly Cross-Adsorbed Secondary Antibody, Alexa Fluor 594, #A21203	IF	1:400

WB, western blot; IF, immunofluorescence.

## Supplementary Materials

Supplementary materials can be found at https://www.mdpi.com/1422-0067/21/12/4563/s1. Supplementary Figure S1. Raw immunoblots.

## Abbreviations

GH	Growth hormone
r-hGH	Recombinant human growth hormone
BrdU	Bromodeoxyuridine
NeuN	Neuronal nuclei
DCX	Doublecortin
GluR1	AMPA Receptor 1
VD	Visual Discrimination
DG	Dentate Gyrus

## References

[1] Prins N.D., van Dijk E.J., den Heijer T., Vermeer S.E., Jolles J., Koudstaal P.J., Hofman A., Breteler M.M. (2005). Cerebral small-vessel disease and decline in information processing speed, executive function and memory. Brain.

[2] Blum S., Luchsinger J.A., Manly J.J., Schupf N., Stern Y., Brown T.R., DeCarli C., Small S.A., Mayeux R., Brickman A.M. (2012). Memory after silent stroke: Hippocampus and infarcts both matter. Neurology.

[3] Gemmell E., Bosomworth H., Allan L., Hall R., Khundakar A., Oakley A.E., Deramecourt V., Polvikoski T.M., O’Brien J.T., Kalaria R.N. (2012). Hippocampal neuronal atrophy and cognitive function in delayed poststroke and aging-related dementias. Stroke.

[4] Selnes P., Grambaite R., Rincon M., Bjørnerud A., Gjerstad L., Hessen E., Auning E., Johansen K., Almdahl I.S., Due-Tønnessen P. (2015). Hippocampal complex atrophy in poststroke and mild cognitive impairment. J. Cereb. Blood Flow Metab..

[5] Khlif M.S., Werden E., Egorova N., Boccardi M., Redolfi A., Bird L., Brodtmann A. (2019). Assessment of longitudinal hippocampal atrophy in the first year after ischemic stroke using automatic segmentation techniques. Neuroimage Clin..

[6] Yagita Y., Kitagawa K., Ohtsuki T., Takasawa K., Miyata T., Okano H., Hori M., Matsumoto M. (2001). Neurogenesis by progenitor cells in the ischemic adult rat hippocampus. Stroke.

[7] Koh S.-H., Park H.-H. (2017). Neurogenesis in Stroke Recovery. Transl. Stroke Res..

[8] Takagi Y., Nozaki K., Takahashi J., Yodoi J., Ishikawa M., Hashimoto N. (1999). Proliferation of neuronal precursor cells in the dentate gyrus is accelerated after transient forebrain ischemia in mice. Brain Res..

[9] Kee N.J., Preston E., Wojtowicz J.M. (2001). Enhanced neurogenesis after transient global ischemia in the dentate gyrus of the rat. Exp. Brain Res..

[10] Tonchev A.B., Yamashima T., Zhao L., Okano H. (2003). Differential proliferative response in the postischemic hippocampus, temporal cortex, and olfactory bulb of young adult macaque monkeys. Glia.

[11] Liu J., Solway K., Messing R.O., Sharp F.R. (1998). Increased neurogenesis in the dentate gyrus after transient global ischemia in gerbils. J. Neurosci..

[12] Lichtenwalner R.J., Parent J.M. (2006). Adult neurogenesis and the ischemic forebrain. J. Cereb. Blood Flow Metab..

[13] Kernie S.G., Parent J.M. (2010). Forebrain neurogenesis after focal Ischemic and traumatic brain injury. Neurobiol. Dis..

[14] Cuartero M.I., de la Parra J., Pérez-Ruiz A., Bravo-Ferrer I., Durán-Laforet V., García-Culebras A., García-Segura J.M., Dhaliwal J., Frankland P.W., Lizasoain I. (2019). Abolition of aberrant neurogenesis ameliorates cognitive impairment after stroke in mice. J. Clin. Investig..

[15] Arvidsson A., Collin T., Kirik D., Kokaia Z., Lindvall O. (2002). Neuronal replacement from endogenous precursors in the adult brain after stroke. Nat. Med..

[16] Parent J.M., Vexler Z.S., Gong C., Derugin N., Ferriero D.M. (2002). Rat forebrain neurogenesis and striatal neuron replacement after focal stroke. Ann. Neurol..

[17] Jin K., Sun Y., Xie L., Peel A., Mao X.O., Batteur S., Greenberg D.A. (2003). Directed migration of neuronal precursors into the ischemic cerebral cortex and striatum. Mol. Cell. Neurosci..

[18] Zhang R., Zhang Z., Wang L., Wang Y., Gousev A., Zhang L., Ho K.L., Morshead C., Chopp M. (2004). Activated neural stem cells contribute to stroke-induced neurogenesis and neuroblast migration toward the infarct boundary in adult rats. J. Cereb. Blood Flow Metab..

[19] Bernal G.M., Peterson D.A. (2004). Neural stem cells as therapeutic agents for age-related brain repair. Aging Cell.

[20] Niv F., Keiner S., Krishna K., Witte O.W., Lie D.C., Redecker C. (2012). Aberrant neurogenesis after stroke: A retroviral cell labeling study. Stroke.

[21] Woitke F., Ceanga M., Rudolph M., Niv F., Witte O.W., Redecker C., Kunze A., Keiner S. (2017). Adult hippocampal neurogenesis poststroke: More new granule cells but aberrant morphology and impaired spatial memory. PLoS ONE.

[22] Bianchi V.E., Locatelli V., Rizzi L. (2017). Neurotrophic and Neuroregenerative Effects of GH/IGF1. Int. J. Mol. Sci..

[23] Aberg N.D., Brywe K.G., Isgaard J. (2006). Aspects of growth hormone and insulin-like growth factor-I related to neuroprotection, regeneration, and functional plasticity in the adult brain. ScientificWorldJournal.

[24] Ong L.K., Chow W.Z., TeBay C., Kluge M., Pietrogrande G., Zalewska K., Crock P., Åberg N.D., Bivard A., Johnson S.J. (2018). Growth Hormone Improves Cognitive Function After Experimental Stroke. Stroke.

[25] Sanchez-Bezanilla S., Åberg N.D., Crock P., Walker F.R., Nilsson M., Isgaard J., Ong L.K. (2020). Growth Hormone Promotes Motor Function after Experimental Stroke and Enhances Recovery-Promoting Mechanisms within the Peri-Infarct Area. Int. J. Mol. Sci..

[26] Heredia M., Palomero J., de la Fuente A., Criado J.M., Yajeya J., Devesa J., Devesa P., Vicente-Villardón J.L., Riolobos A.S. (2018). Motor Improvement of Skilled Forelimb Use Induced by Treatment with Growth Hormone and Rehabilitation Is Dependent on the Onset of the Treatment after Cortical Ablation. Neural Plast..

[27] Heredia M., Rodríguez N., Sánchez Robledo V., Criado J.M., de la Fuente A., Devesa J., Devesa P., Sánchez Riolobos A. (2019). Factors Involved in the Functional Motor Recovery of Rats with Cortical Ablation after GH and Rehabilitation Treatment: Cortical Cell Proliferation and Nestin and Actin Expression in the Striatum and Thalamus. Int. J. Mol. Sci..

[28] Heredia M., Fuente A., Criado J., Yajeya J., Devesa J., Riolobos A.S. (2013). Early growth hormone (GH) treatment promotes relevant motor functional improvement after severe frontal cortex lesion in adult rats. Behav. Brain Res..

[29] Pathipati P., Surus A., Williams C.E., Scheepens A. (2009). Delayed and chronic treatment with growth hormone after endothelin-induced stroke in the adult rat. Behav. Brain Res..

[30] Horner A.E., Heath C.J., Hvoslef-Eide M., Kent B.A., Kim C.H., Nilsson S.R.O., Alsiö J., Oomen C.A., Holmes A., Saksida L.M. (2013). The touchscreen operant platform for testing learning and memory in rats and mice. Nat. Protoc.

[31] Sanchez-Bezanilla S., TeBay C., Nilsson M., Walker F.R., Ong L.K. (2019). Visual discrimination impairment after experimental stroke is associated with disturbances in the polarization of the astrocytic aquaporin-4 and increased accumulation of neurotoxic proteins. Exp. Neurol..

[32] Baumgartner P., El Amki M., Bracko O., Luft A.R., Wegener S. (2018). Sensorimotor stroke alters hippocampo-thalamic network activity. Sci. Rep..

[33] Ong L.K., Walker F.R., Nilsson M. (2017). Is Stroke a Neurodegenerative Condition? A Critical Review of Secondary Neurodegeneration and Amyloid-beta Accumulation after Stroke. AIMS Med. Sci..

[34] Zhang J., Zhang Y., Xing S., Liang Z., Zeng J. (2012). Secondary neurodegeneration in remote regions after focal cerebral infarction: A new target for stroke management?. Stroke.

[35] Jin K., Minami M., Lan J.Q., Mao X.O., Batteur S., Simon R.P., Greenberg D.A. (2001). Neurogenesis in dentate subgranular zone and rostral subventricular zone after focal cerebral ischemia in the rat. Proc. Natl. Acad. Sci. USA.

[36] Feng X., Li G., Wu W., Xu Y., Lin H., Fan J. (2020). Recombinant Human Growth Hormone Ameliorates Cognitive Impairment in Stroke Patients. J. Comput. Assist. Tomogr..

[37] Nyberg F., Hallberg M. (2013). Growth hormone and cognitive function. Nat. Rev. Endocrinol..

[38] Pan W., Yu Y., Cain C.M., Nyberg F., Couraud P.O., Kastin A.J. (2005). Permeation of growth hormone across the blood-brain barrier. Endocrinology.

[39] López-Fernández J., Sánchez-Franco F., Velasco B., Tolón R.M., Pazos F., Cacicedo L. (1996). Growth hormone induces somatostatin and insulin-like growth factor I gene expression in the cerebral hemispheres of aging rats. Endocrinology.

[40] Ye P., Umayahara Y., Ritter D., Bunting T., Auman H., Rotwein P., D’Ercole A.J. (1997). Regulation of insulin-like growth factor I (IGF-I) gene expression in brain of transgenic mice expressing an IGF-I-luciferase fusion gene. Endocrinology.

[41] Reinhardt R.R., Bondy C.A. (1994). Insulin-like growth factors cross the blood-brain barrier. Endocrinology.

[42] Armstrong C.S., Wuarin L., Ishii D.N. (2000). Uptake of circulating insulin-like growth factor-I into the cerebrospinal fluid of normal and diabetic rats and normalization of IGF-II mRNA content in diabetic rat brain. J. Neurosci. Res..

[43] Brown J.P., Couillard-Després S., Cooper-Kuhn C.M., Winkler J., Aigner L., Kuhn H.G. (2003). Transient expression of doublecortin during adult neurogenesis. J. Comp. Neurol..

[44] Pekna M., Pekny M., Nilsson M. (2012). Modulation of neural plasticity as a basis for stroke rehabilitation. Stroke.

[45] Hollmann M., Heinemann S. (1994). Cloned glutamate receptors. Annu. Rev. Neurosci..

[46] Dingledine R., Borges K., Bowie D., Traynelis S.F. (1999). The glutamate receptor ion channels. Pharmacol. Rev..

[47] Lu W., Isozaki K., Roche K.W., Nicoll R.A. (2010). Synaptic targeting of AMPA receptors is regulated by a CaMKII site in the first intracellular loop of GluA1. Proc. Natl. Acad. Sci. USA.

[48] Chater T.E., Goda Y. (2014). The role of AMPA receptors in postsynaptic mechanisms of synaptic plasticity. Front. Cell. Neurosci..

[49] Mustafa A., Adem A., Roos P., Nyberg F. (1994). Sex differences in binding of human growth hormone to rat brain. Neurosci. Res..

[50] Furigo I.C., Metzger M., Teixeira P.D., Soares C.R., Donato J. (2017). Distribution of growth hormone-responsive cells in the mouse brain. Brain Struct. Funct..

[51] Nilsson A., Carlsson B., Isgaard J., Isaksson O.G., Rymo L. (1990). Regulation by GH of insulin-like growth factor-I mRNA expression in rat epiphyseal growth plate as studied with in-situ hybridization. J. Endocrinol..

[52] Walser M., Schiöler L., Oscarsson J., Åberg M.A., Wickelgren R., Svensson J., Isgaard J., Åberg N.D. (2017). Mode of GH administration and gene expression in the female rat brain. J. Endocrinol..

[53] Walser M., Schiöler L., Oscarsson J., Aberg M.A., Svensson J., Aberg N.D., Isgaard J. (2014). Different modes of GH administration influence gene expression in the male rat brain. J. Endocrinol..

[54] Song J., Park K., Lee H., Kim M. (2012). The effect of recombinant human growth hormone therapy in patients with completed stroke: A pilot trial. Ann. Rehabil. Med..

[55] Lillicrap T., Garcia-Esperon C., Walker F.R., Ong L.K., Nilsson M., Spratt N., Levi C.R., Parsons M., Isgaard J., Bivard A. (2018). Growth Hormone Deficiency Is Frequent After Recent Stroke. Front. Neurol.

[56] Kreber L.A., Ashley M.J., Masel B.E., Singh C.K., Randle K.D., Johnson C., Helvie R., Ashley M.J., Griesbach G.S. (2020). Prevalence of growth hormone deficiency in middle-age adults recovering from stroke. Brain Inj..

[57] Zalewska K., Ong L.K., Johnson S.J., Nilsson M., Walker F.R. (2017). Oral administration of corticosterone at stress-like levels drives microglial but not vascular disturbances post-stroke. Neuroscience.

[58] Zhao Z., Ong L.K., Johnson S., Nilsson M., Walker F.R. (2017). Chronic stress induced disruption of the peri-infarct neurovascular unit following experimentally induced photothrombotic stroke. J. Cereb. Blood Flow Metab..

[59] Walser M., Hansén A., Svensson P.-A., Jernås M., Oscarsson J., Isgaard J., Åberg N.D. (2011). Peripheral administration of bovine GH regulates the expression of cerebrocortical beta-globin, GABAB receptor 1, and the Lissencephaly-1 protein (LIS-1) in adult hypophysectomized rats. Growth Horm. IGF Res..

[60] Kluge M.G., Abdolhoseini M., Zalewska K., Ong L.K., Johnson S.J., Nilsson M., Walker F.R. (2019). Spatiotemporal analysis of impaired microglia process movement at sites of secondary neurodegeneration post-stroke. J. Cereb. Blood Flow Metab..

[61] Kluge M.G., Kracht L., Abdolhoseini M., Ong L.K., Johnson S.J., Nilsson M., Walker F.R. (2017). Impaired microglia process dynamics post-stroke are specific to sites of secondary neurodegeneration. Glia.

[62] Pietrogrande G., Zalewska K., Zhao Z., Abdolhoseini M., Chow W.Z., Sanchez-Bezanilla S., Ong L.K., Johnson S.J., Nilsson M., Walker F.R. (2019). Low oxygen post conditioning prevents thalamic secondary neuronal loss caused by excitotoxicity after cortical stroke. Sci. Rep..

[63] Ong L.K., Zhao Z., Kluge M., Walker F.R., Nilsson M. (2017). Chronic stress exposure following photothrombotic stroke is associated with increased levels of Amyloid beta accumulation and altered oligomerisation at sites of thalamic secondary neurodegeneration in mice. J. Cereb. Blood Flow Metab..

[64] Sanchez-Bezanilla S., Nilsson M., Walker F.R., Ong L.K. (2019). Can We Use 2,3,5-Triphenyltetrazolium Chloride-Stained Brain Slices for Other Purposes? The Application of Western Blotting. Front. Mol. Neurosci..

