# Supplementary material for: Growth Hormone Treatment Promotes Remote Hippocampal Plasticity after Experimental Cortical Stroke
Source: Int J Mol Sci. 2020 Jun 26;21(12):4563. doi: 10.3390/ijms21124563 (PMC7349868; doi:10.3390/ijms21124563)
Supplement: Supplementary file 1 [file ijms-21-04563-s001.pdf]

International Journal of
Molecular Sciences

Supplementary Materials

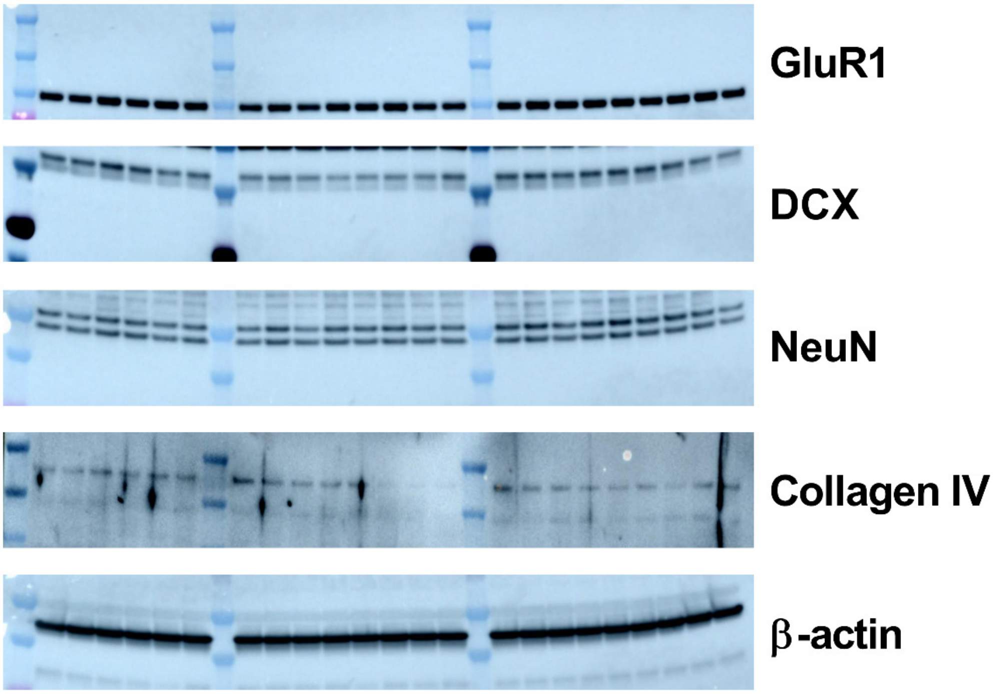

Figure S1. Raw immuno 10.3390/ijms21124563 blots corresponding to GluR1, DCX, NeuN, Collagen IV and β-actin.
